# Systemic associations of pyoderma gangrenosum: a systematic review

**DOI:** 10.1093/skinhd/vzag037

**Published:** 2026-05-26

**Authors:** Nageswary Nadarajah, Cedric Ho Tiu, Shernaz Walton

**Affiliations:** Department of Dermatology, Leeds Teaching Hospitals NHS Trust, Leeds, UK; Department of Dermatology, Aberdeen Royal Infirmary, Aberdeen, UK; University of Hull, Hull University Teaching Hospitals NHS Foundation Trust, Hull, UK

## Abstract

**Background:**

Pyoderma gangrenosum (PG) is a rare neutrophilic dermatosis that may be associated with systemic diseases. Characterizing these associations is clinically relevant for diagnosis, prognosis and management.

**Objectives:**

To systematically review acquired systemic diseases associated with PG in adults and describe related clinical features and treatment approaches.

**Methods:**

We searched PubMed/MEDLINE, Embase and Scopus from January 2015 to May 2025. Eligible studies included observational studies, case reports and case series reporting systemic associations of PG in adults. Data extraction was performed independently by two reviewers and the risk of bias was assessed using the Joanna Briggs Institute checklists.

**Results:**

Nineteen observational studies and 18 case-based studies were included. The most frequent systemic associations were inflammatory bowel disease (*n* = 13), haematological disorders (*n* = 6), solid organ malignancy (*n* = 3) and arthritis (*n* = 3). Less common associations included vasculitides, autoimmune conditions, connective tissue diseases and organ-­specific disorders. In the observational literature, only three studies reported PG subtypes, with the ulcerative form accounting for more than 85% of cases. Case-based literature highlighted patterns such as relapse-prone disease in patients with vasculitis and therapeutic responses to biologics in refractory cases.

**Conclusions:**

PG demonstrates diverse systemic associations, ranging from well-established links to rarer conditions. Observational studies strengthen evidence for common associations, while case-based literature provides insights into clinical patterns and emerging therapies. Larger prospective studies are needed to clarify causality and optimize management.

## Teaser

Pyoderma gangrenosum is a rare neutrophilic dermatosis that may be associated with systemic diseases. Characterizing these associations is clinically relevant for diagnosis, prognosis and management.

What is already known about this topic?

- Pyoderma gangrenosum (PG) is a rare neutrophilic dermatosis that is frequently associated with systemic diseases.
- The most well-established links are with inflammatory bowel disease and haematological disorders.
- Evidence for rarer systemic associations is limited to case reports and case series.

What does this study add?

- This review systematically synthesizes evidence on systemic associations of PG, focusing on literature published since 2015.
- It highlights established and emerging systemic links, including rarer autoimmune, connective tissue, vasculitic and ­infectious conditions.
- It provides insights into timing of associations, clinical patterns and evidence gaps, guiding future research and improving patient care.

Pyoderma gangrenosum (PG) is a rare neutrophilic dermatosis, with an estimated annual incidence of 3–10 cases per million population, and is more prevalent in female individuals.^1^ The classical presentation is a sterile papule or pustule that rapidly evolves into a painful ulcer with a raised, undermined, violaceous and inflamed border. Lesions may be precipitated by minor trauma, a phenomenon known as pathergy. The ulcerative subtype accounts for approximately 85% of cases, while bullous, pustular and vegetative ­phenotypes are less common. Although histological findings are nonspecific, biopsy is useful to exclude alternative causes of ulceration such as vascular occlusive or venous disease, vasculitis, cancer, primary infection and drug-induced ulcers.^2^ Diagnosis is therefore primarily clinical. To support diagnosis, several diagnostic and scoring criteria have been proposed, including those by Su *et al.*, the Delphi consensus and the PARACELSUS score.^3–5^ Haag *et al.* reported that the PARACELSUS score identified the highest proportion of patients with PG (89%), compared with 74% for the Delphi and Su criteria.^6^ The morbidity and mortality of PG is high; therefore, early and accurate diagnosis are crucial.^7^

The exact pathogenesis of PG remains unclear. It appears to involve a complex interplay between genetic predisposition, autoinflammatory syndromic variants, and dysregulation of innate and adaptive immunity.^4^ The association of PG with systemic diseases has been reported to be as high as 80%, while a meta-analysis from 2018 reported pooled estimates of 56.8%.^8,9^ The most frequent systemic associations were inflammatory bowel disease (IBD; 17.6%), arthritis (12.8%), haematological malignancies (8.9%) and solid malignancies (7.4%).^9^

First-line treatment typically involves a fast-acting immunosuppressant such as systemic corticosteroids or ciclosporin, often followed by a steroid-sparing agent. Adjunctive wound care and optimization of healing factors are also essential.^4,10,11^ Due to the rarity of PG, evidence-based treatment remains limited, with only two randomized controlled trials published to date.^12,13^

The aim of this review is to synthesize the latest literature with regard to systemic associations of PG, to identify new associations, to explore the related clinical patterns and to summarize their management strategies.

## Materials and methods

### Protocol and registration

This systematic review was conducted in accordance with the PRISMA 2020 guidelines. The protocol was prospectively registered with PROSPERO (registration number CRD420251081307) and is available at https://www.crd.york.ac.uk/PROSPERO/view/CRD420251081307.

### Eligibility criteria

We included studies involving adults (aged≥18 years) diagnosed with PG, as determined by clinical features, histopathology or recognized diagnostic criteria. Studies were eligible if they reported one or more acquired systemic diseases associated with PG, occurring before or concurrently with or identified during the diagnostic investigations for PG. Systemic diseases were grouped into broad, non-mutually exclusive categories for synthesis, including ­inflammatory, autoimmune, vasculitic, infectious, neoplastic, haematological (malignant and nonmalignant) and metabolic conditions. Metabolic conditions included disorders such as diabetes mellitus, thyroid dysfunction and sarcoidosis, where systemic inflammation or immune dysregulation may be implicated.

We excluded studies involving paediatric patients (aged <18 years) and those in which systemic diseases were diagnosed several years after PG onset without a clear temporal or mechanistic link. We also excluded studies where PG diagnosis was unclear, misclassified or not clinically described. We excluded studies of inherited autoinflammatory syndromes, including pyogenic arthritis, pyoderma gangrenosum and acne syndrome (PAPA); pyoderma gangrenosum, acne and hidradenitis suppurativa syndrome (PASH); pyogenic arthritis, pyoderma gangrenosum, acne and hidradenitis suppurativa syndrome (PAPASH); PSTPIP1-associated myeloid-related proteinemia inflammatory syndrome (PAMI); pyoderma gangrenosum, acne, hidradenitis suppurativa and seronegative spondyloarthritis syndrome (PASS); and trauma-­induced PG in the absence of systemic disease (idiopathic postsurgical PG and peristomal PG). Additionally, we excluded drug-­induced PG, PG triggered by HIV (particularly in immune reconstitution inflammatory syndrome), pregnancy and venous insufficiency.

No comparators or control groups were required. Eligible study designs included observational studies (cohort, case–control, cross-sectional), registry/database studies and clinical trials if relevant baseline or outcome data on systemic features were reported. Case reports (*n* = 1–2) and case series (*n* ≥ 3) describing PG in association with systemic diseases that are under-represented in observational studies were eligible for inclusion, in order to capture treatment approaches in clinical contexts where higher-level evidence remains limited. We excluded editorials, commentaries, letters, conference abstracts without data, qualitative studies, basic science research and publications not written in English. Systematic reviews and narrative reviews were excluded from analysis but were screened for eligible primary studies through reference list cross-checking.

### Information sources

We systematically searched PubMed/MEDLINE, Embase and Scopus on 24 May 2025. Additional references were identified by reviewing the bibliographies of systematic reviews and meta-analyses.

### Search strategy

Search strategies were developed using a combination of medical subject heading (MeSH) terms and text words related to ‘pyoderma gangrenosum’ and various systemic diseases (IBD, arthritis and haematological disorders, among others). The full search strategy for each database is provided in Table S1 (see Supporting Information).

### Selection process

**Figure 1:**
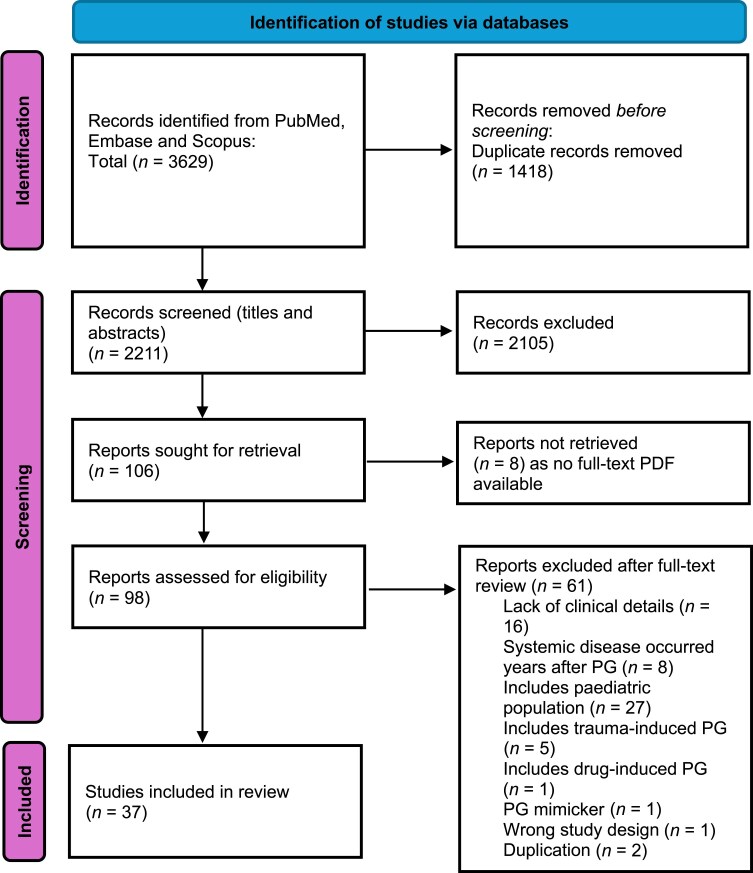
PRISMA 2020 flow diagram detailing the study selection process. PG, pyoderma gangrenosum.

The study selection process is outlined in the PRISMA 2020 flow diagram (Figure 1). All titles and abstracts were independently screened by two reviewers (N.N. and C.H.T.) using the online platform Rayyan. Full-text screening was then conducted for all potentially relevant articles. Discrepancies were resolved through discussion or by consulting a third reviewer (S.W.), a clinical expert in PG.

### Data collection process

Data were independently extracted by two reviewers (N.N. and C.H.T.) using a standardized extraction template developed beforehand. Discrepancies were discussed and resolved through consensus. Data extraction focused on identifying systemic diseases associated with PG, their temporal relationship to PG onset and relevant clinical features of PG such as treatment and outcomes.

### Data items

For observational studies, the following data were extracted: first author, year, country, study design, PG sample size, associated systemic disease(s), category of systemic disease, timing of association relative to PG, disease activity at PG onset, key findings, novelty and risk of bias. For case-based studies, additional items extracted included patient age and sex, ulcer characteristics, PG subtype (if reported), site of involvement, presence of pathergy, recurrence, treatment and clinical outcome.

### Risk of bias assessment

Risk of bias was assessed independently by two reviewers (N.N. and C.H.T.) using the appropriate Joanna Briggs Institute (JBI) critical appraisal checklists. Specific tools were used for observational studies, case reports and case series. Each item was marked as ‘Yes’, ‘No’, ‘Unclear’ or ‘Not applicable’, and discrepancies were resolved by discussion. A summary of the risk of bias assessment is presented in Tables S2 and S3 (see Supporting Information). JBI checklists used for risk of bias assessments are available upon request from the corresponding author.

### Effect measures

Given the heterogeneity of study designs and reporting, a meta-analysis was not performed. For observational studies that reported quantitative measures [hazard ratios (HRs) and odds ratios (ORs)], we extracted and presented the effect estimates along with their 95% confidence intervals (CIs).

### Synthesis methods

A narrative synthesis was performed where observational studies were summarized in a table addressing the primary objective: systemic associations of PG and their temporal relationship (Table 1; Table S2). Case-based studies were summarized in a second table addressing secondary objectives, including ulcer characteristics, treatment and outcomes. Findings were grouped by systemic disease category (Tables 1, 2; Tables S2, S3).

**Table 1:** Summary of observational studies included in the systematic review

Study	Design	Population studied	Control	Disease association	Key findings
Schosler, 2021 (Denmark)^14^	Retrospective cohort study	PG (*n* = 64)	No	Multiple	IBD (28.1%), type 2 diabetes (21.9%), cardiac (20.3%), malignancy after PG (14.1%), RA (12.5%), thyroid disease (12.5%), other autoimmune diseases (12.5%), gout (7.8%), haematological (7.8%), malignancy before PG (6.3%), kidney disease (4.7%), HS (4.7%), type 1 diabetes (3.1%), parkinsonism (3.1%), haemophagocytosis (1.6%). Types of PG: ulcerative (85.9%), peristomal (6.3%), bullous (3.1%), vegetative (3.1%), pustular (1.6%)
Ben Abdallah, 2021 (Denmark)^7^	Nationwide registry nested case–control study	PG (*n* = 1604)	Yes, *n* = 16 039	Multiple	1023 of 1604 with PG had a comorbidity (64%) vs. 33% in the control group. Comorbidities in patients with PG compared with control individuals with *P* <0.0025: diabetes without complication, heart failure or cardiomyopathy, hemiplegia or paraplegia, hidradenitis or acne, lymphoma, IBD, leukaemia, mild liver disease, moderate-to-severe renal disease, osteoporosis, peripheral vascular disease, rheumatic disease
Vacas, 2017 (Argentina)^15^	Retrospective cohort study	PG (*n* = 31)	No	Multiple	23 of 31 with PG (74%) had associated systemic disease: IBD 10 (32%), haematological malignancies 7 (22%), rheumatoid or seronegative arthritis 5 (16%), diabetes 5 (16%), cocaine consumption 1 (3%)
Kaffenberger, 2018 (USA)^16^	Retrospective cross-sectional study	PG (*n* = 31 885)	No	Multiple	Comorbidities: IBD 8453 (26.5%), inflammatory arthritis 2345 (7.35%), vasculitis and HSP 795 (2.49%), haematological malignancy and dyscrasia 886 (2.78%); others 19 405 (60.86%)
Ashchyan, 2018 (USA)^17^	Retrospective cohort	PG (*n* = 356)	No	Multiple	238 of 356 with PG (66.9%) had comorbidities: IBD: 146 patients (41%) [CD 92 (25.8%), UC 55 (15.4%)], arthritis: 73 (20.5%), malignant neoplasms: 44 (12.4%) [solid organ malignancies 23 (6.5%) and haematological malignancies 21 (5.9%)], haematological disorders (nonmalignant): 17 (4.8%). Age associations: IBD was significantly more common in patients aged <65 years (*P* < 0.001); and arthritis, solid organ malignancies and haematological disorders (including malignancies) were more common in patients aged ≥65 years (*P* < 0.05)
Saeidi, 2024 (Canada)^18^	Retrospective cohort study	PG (*n* = 106)	No	MG (haematological)	29 of 106 with PG (27%) had MG. Most common subtype: IgA (41%), IgG (28%) and biclonal IgA/IgG (14%). Cancer developed in significantly more patients with PG with gammopathy than without (28% vs. 6%, *P* = 0.003), predominantly haematological malignancies (especially multiple myeloma). Among subtypes, IgG MG had the highest proportion of cancer (50%)
Kridin, 2021 (Israel)^19^	Retrospective cohort + case–control	PG (*n* = 302)	Yes, *n* = 1497	SOM	No significant bidirectional association between PG and SOM was found. Risk of SOM after PG: aHR 1.02 (95% CI 0.52–2.02); risk of PG after SOM: aOR 0.85 (95% CI 0.51–1.41; *P* = 0.522). Patients with coexistent PG and SOM were significantly older at PG onset compared with those with PG alone: mean (SD) age 71.7 (12.2) years vs. 51.9 (20.7) years (*P* < 0.001)
Kridin, 2021 (Israel)^20^	Retrospective cohort + case–control	PG (*n* = 302)	Yes, *n* = 1497	CRC	A bidirectional association was observed between PG and several renal comorbidities. In the retrospective cohort analysis, patients with PG had an increased risk of CRF (aHR 3.68; 95% CI 2.72–5.97); dialysis (aHR 27.79; 95% CI 3.24–238.14); OKD (aHR 2.71; 95% CI 1.55–4.74); no significant association with KT. In the case–control analysis, patients with prior renal comorbidities had higher odds of PG: CRF (aOR 2.34; 95% CI 1.33–4.11); KT (aOR 5.03; 95% CI 1.01–25.12); OKD (aOR 1.69; 95% CI 1.04–2.74); no significant association for prior dialysis. Patients with PG and CRC were older than those without CRC (mean age >60 years, *P* < 0.05)
Juliao-Baños, 2021 (Colombia)^21^	Retrospective cohort	IBD (*n* = 744)	No	PG	8 of 744 with IBD (1.1%) had PG: 7 of 544 with UC (1.2%) and 1 of 200 with CD (0.5%). No effect estimate provided; descriptive prevalence only
Jonaitytė, 2024 (Lithuania)^22^	Prospective cross-sectional	IBD (*n* = 162)	No	PG	6 of 162 with IBD (3.7%) had PG: with 5 of 117 UC (4.3%) and 1 of 45 CD (2.2%). No statistically significant difference between UC and CD (*P* = 0.877). No association with IBD duration (*P* > 0.05)
Ghani, 2024 (Pakistan)^23^	Retrospective cross-sectional study	IBD (*n* = 250)	No	PG	14 of 250 with IBD (5.6%) had PG; no significant difference between CD (6.4%) and UC (4.5%) (*P* = 0.45). PG significantly more common in active disease (45%) than remission (18%) (*P* < 0.001)
Waljee, 2020 (USA)^24^	Retrospective cross-sectional study	Single centre: IBD (*n* = 6225); national: IBD (*n* = 80 907)	Yes. Single centre: *n* = 31 125; national: *n* = 404 535	PG	Single centre: 44 of 6225 IBD (0.71%) had PG, 10 of 31 125 controls (0.03%) had PG; OR 22.15, *P* = 0.0001. National: 607 of 80 907 IBD (0.8%) had PG, 492 of 404 535 control individuals (0.1%) had PG; OR 6.2, *P* = 0.0001
Giraudo, 2021 (Argentina)^25^	Retrospective observational study	IBD (*n* = 444)	No	PG	7 of 444 had PG (1.6%): 5 had established IBD, 2 were concomitantly diagnosed with IBD and PG. One patient developed PG 1 year after colectomy
Halling, 2017 (Denmark)^26^	Nationwide retrospective cross-sectional study	IBD (*n* = 47 325)	Yes, *n* = 92 839	PG	193 of 47 325 with IBD had PG, with OR 47.5, 95% CI (23.4–96.4); 8 of the 92 839 controls had PG
Padhi, 2023 (India)^27^	Prospective cohort study	UC (*n* = 112)	Yes, *n* = 100	PG	1 of 112 with UC (0.9%) had PG, which disappeared within a month of treatment of the UC
Yang, 2018 (Korea)^28^	Nationwide retrospective cross-sectional study	CD (*n* = 13 925); UC (*n* = 29 356)	Yes, *n* = 1 127 261	PG	32 of 13 925 with CD (0.2%) had PG. 34 of 29 356 with UC (0.1%) had PG; 341 of 1 127 261 control individuals (0.0%) had PG. Standardized prevalence ratio (95% CI) for the CD group was 4.43 (1.36–7.50) and the UC group was 4.36 (2.50–6.23)
Lee, 2016 (Korea)^29^	Retrospective cohort study	MDS with AIMs (*n* = 67)	Yes, *n* = 134 MDS without AIMs	PG	2 of 67 with MDS had PG
Bugaut, 2023 (France)^30^	Retrospective cohort study	Behçets (*n* = 20)	No	PG	6 of 20 with Behçets (30%) had PG and all involved the lower limbs
Frumholtz, 2017 (France)^31^	Retrospective cohort	AAV (*n* = 1553) (vasculitis)	No	PG	8 of 1553 with AAV had PG and they were all GPA; nil in EGPA and MPA, *P* = 0.01

AAV, ANCA-associated vasculitides; adj, adjusted; AIMs, autoimmune manifestations; CD, Crohn disease; CI, confidence interval; CRC, chronic renal comorbidities; CRF, chronic renal failure, EGPA, eosinophilic granulomatosis with polyangiitis; GPA, granulomatosis with polyangiitis; HR, hazard ratio; HS, hidradenitis suppurativa; HSP, Henoch–Schönlein purpura; IBD, inflammatory bowel disease; KT, kidney transplantation; MDS, myelodysplastic syndrome; MG, monoclonal gammopathy; MPA, microscopic polyangiitis; OKD, other kidney diseases; OR, odds ratio; PG, pyoderma gangrenosum; RA, rheumatoid arthritis; SOM, solid organ malignancy; UC, ulcerative colitis.

**Table 2:** Summary of case reports and case series included in the systematic review

Study	No. of patients	Age (years), sex	Systemic disease	Systemic disease category	Timing of systemic disease diagnosis relative to PG	Site(s) of involvement	PG subtypes	Treatment + outcome
Chua, 2018 (UK) (case report)^32^	1	49, F	Atypical Cogan syndrome	Vasculitis	Concurrent	Both shins	Not reported	Multiple therapies failed; good response to CYC
Nadarajah, 2025 (UK) (case report)^33^	1	75, F	Atypical Cogan syndrome	Vasculitis	Before	R buttock, L hip and buttock	Not reported	Good response to corticosteroids + CYC + hyperbaric oxygen therapy
de Boysson, 2016 (France) (case series)^34^	8	30–62, 5 M, 3F	GPA	Vasculitis	4 concurrent, 2 before PG, 2 after PG	Abdomen, lower limb, trunk, back	Not reported	All responded to corticosteroids ± immunosuppressants (e.g. CYC, AZA, IVIg, CsA)
Malinowska, 2020 (Poland) (case report)^35^	1	41, M	GPA	Vasculitis	Concurrent	L shin	Not reported	Partial response to corticosteroids + CsA + pentoxifylline; good response to corticosteroids + CYC, with AZA for maintenance
Barrera-Vargas 2015 (Mexico) (case report)^36^	2	21 and 26, 1 M, 1F	Takayasu arteritis	Vasculitis	(i) 5 years after PG	(i) Head, neck, arms, lower limbs	Not reported	(i) Partial response to prednisone, thalidomide
					(ii) 8 years after PG			(ii) Good response to prednisone, CsA, thalidomide, dapsone, AZA
Bhowmick, 2023 (India) (case report)^37^	1	22 F	Takayasu arteritis	Vasculitis	Concurrent	Bilateral upper limbs and lower limbs	Not reported	Trialled corticosteroids, MTX, colchicine, tocilizumab, antibiotics; responded only to tofacitinib
Loetscher, 2016 (Switzerland) (case report)^38^	1	43 F	Takayasu arteritis	Vasculitis	Concurrent	Bilateral lower limbs	Not reported	Good response to corticosteroids + CsA + MTX
Ozuguz, 2015 (Turkey) (case report)^39^	1	33 F	Behçets	Autoinflammatory/autoimmune	Concurrent	R labium minor	Vegetative	No response to colchicine, good response to corticosteroids
Pourbagherian, 2023 (Iran) (case report)^40^	1	55 F	SLE	Autoimmune	Before PG by 20 years	Anterior and lateral R leg	Not reported	Good response to corticosteroids + CYC + hydroxychloroquine + mycophenolate mofetil
Lebrun 2018 (France) (case report)^41^	2	32 and 37, 2 F	SLE	Autoimmune	(i) 1 year before PG	(i) Inner canthus L eye, L corner of lips, cervical	Ulcerative for both	(i) Complete response to corticosteroids
					(ii) 10 years before PG	(ii) Posterior legs		(ii) Failure (side effects) with dapsone, colchicine, low-dose corticosteroid. Complete response with corticosteroids and MTX
Frioui, 2022 (Tunisia) (case report)^42^	1	52 F	Coeliac disease	Autoimmune	Concurrent	Anterior L shin	Ulcerative	Good response to corticosteroids
Yang, 2018 (Taiwan) (case report)^43^	1	33 M	Evans syndrome	Autoimmune	7 years before PG	Scrotum	Ulcerative	Complete response to IV methylprednisolone, then prednisolone and AZA
Dantas, 2017 (Brazil) (case report)^44^	1	25 F	Autoimmune hepatitis	Autoimmune	Concurrent	Bilateral lower limbs	Ulcerative	Complete response with prednisolone; topical noncorticosteroid treatment and dressings
Androutsakos, 2015 (Greece) (case report)^45^	1	19 F	Autoimmune hepatitis	Autoimmune	4 years before PG	Bilateral legs	Not reported	Complete response with prednisolone, CsA, MTX
Kaur, 2022 (India) (case report)^46^	1	43 F	Hepatitis C infection	Infectious	Concurrent	Right lower limb and L buttock	Not reported	Good response to corticosteroids + antibiotics
Skopis, 2021 (USA) (case report)^47^	1	65 F	Limited cutaneous systemic sclerosis	Connective tissue disease	Concurrent	L index finger	Not reported	Complete response to IV methylprednisolone, then prednisolone and topical clobetasol 0.05%
Riyaz, 2015 (India) (case report)^48^	1	27 M	Microscopic colitis, IHES, selective IgE deficiency	Gastrointestinal (non-IBD), haematological, primary immunodeficiency	Concurrent	L lower leg	Not reported	Good response to corticosteroids
Opalińska, 2021 (Poland) (case report)^49^	1	35 M	Haemophagocytic lymphohistiocytosis	Haematological (nonmalignant)	Concurrent	Bilateral upper limbs and lower limbs	Not reported	Multiple systemic therapies trialled (corticosteroids, CsA, IVIg, anakinra); fatal outcome due to sepsis and multiorgan failure

AZA, azathioprine; CsA, ciclosporin; CYC, cyclophosphamide; F, female; GPA, granulomatosis with polyangiitis; IBD, inflammatory bowel disease; IHES, idiopathic hypereosinophilic syndrome; IV, intravenous; IVIg, intravenous immunoglobulins; L, left; M, male; MTX, methotrexate; PG, pyoderma gangrenosum; R, right; SLE, systemic lupus erythematosus.

## Results

### Study selection

A total of 3629 records were identified through database searching. After removing duplicates, 2211 records remained and were screened by title and abstract. Of these, 106 full-text articles were assessed for eligibility. Ultimately, 37 studies met the inclusion criteria and were included in the final review. Reasons for exclusion of full-text articles are detailed in Figure 1.

### Study characteristics

A total of 37 studies were included: 19 observational studies and 18 case-based studies. The observational studies comprised 11 cohort studies (including 2 that also incorporated case–control methodologies), 1 nested case–control study and 7 cross-sectional studies. The case-based studies included 17 single-patient case reports and 1 case series. The studies were published between 2015 and 2025, with most conducted in the USA, France and India. A detailed summary of the studies is provided in Tables 1 and 2 and Tables S2 and S3.

## Summary of results (observational studies)

### Inflammatory bowel disease

Nineteen observational studies explored the systemic associations of PG (Table 1; Table S2). The most frequently investigated associations were with IBD, haematological disorders, solid organ malignancies and arthritis. Less commonly studied associations included vasculitis, Behçets, organ-specific and metabolic conditions.

A total of 13 studies reported associations between PG and IBD, including Crohn disease (CD) and ulcerative colitis (UC). A large national cross-sectional study found PG in 0.8% of patients with IBD compared with 0.1% of control individuals (OR 6.2; *P*<0.0001).^24^ Another population-based study reported significantly increased odds of PG among patients with IBD (OR 47.5; 95% CI 23.4–96.4).^26^ A separate national registry study reported similar elevated standardized prevalence ratios for PG in CD (4.43) and UC (4.36) compared with the general population.^28^ Consistently, two studies found no statistically significant difference in PG prevalence between CD and UC, with *P*-values of 0.45 and 0.877, respectively.^22,23^

Two large US database studies found that IBD was among the most common comorbidities in PG, with prevalence ranging from 26% to 41%, and was significantly more frequent among patients with PG aged < 65 years (*P* < 0.001).^16,17^ In addition, a nationwide Danish registry also reported substantially higher odds of IBD among patients with PG (OR 19.15; 95% CI 15.27–24.02).^7^

Several studies reported a higher prevalence of PG in patients with active IBD compared with those in remission,^23,25^ and one study described resolution of PG following remission in UC.^15^ In most cases, PG was diagnosed during active phases of IBD or shortly after the IBD diagnosis.^15,22,25^

### Haematological disorders

Six studies reported haematological associations with PG, including malignant and nonmalignant disorders (Table 1; Table S2).

Two US database studies identified haematological conditions in 3–6% of patients with PG, with malignancies slightly more prevalent than nonmalignant disorders.^16,17^ One study reported haematological malignancy in 5.9% and nonmalignant haematological disorders in 4.8% of cases.^17^ Another study found haematological malignancy in 2.8% of PG-related hospitalizations, and these patients had a significantly higher risk of in-hospital mortality compared with patients with PG and IBD (OR 4.31; 95% CI 1.78–10.43).^16^

A Canadian cohort study identified monoclonal gammopathy (MG) in 27% of patients with PG, with IgA being the most common subtype (41%). Neoplasms occurred more frequently in patients with PG with MG than those without (28% vs. 6%; *P* = 0.003), predominantly multiple myeloma.^18^

### Solid organ malignancy

The association between PG and solid organ malignancy (SOM) was examined in three studies (Table 1; Table S2).

A US registry study reported SOM in 6.5% of patients with PG.^17^ A Danish cohort study found malignancy preceding PG in 6.3% of cases and malignancy developing after PG in 14.1%.^14^

In contrast, an Israeli retrospective cohort and case–control study found no significant bidirectional association between PG and SOM. The adjusted HR for developing SOM after PG was 1.02 (95% CI 0.52–2.02), and the odds of PG after SOM were also nonsignificant (adjusted OR 0.85, 95% CI 0.51–1.41; *P* = 0.522). Patients with coexistent PG and SOM were also significantly older at PG onset compared with those with PG alone: mean (SD) age 71.7 (12.2) years vs. 51.9 (20.7) years (*P* < 0.001).^19^

### Inflammatory arthritis

The relationship between PG and arthritis was examined in three observational studies (Table 1; Table S2).

A US study found inflammatory arthritis in 7.35% of patients hospitalized with PG. Multivariable logistic regression modelling revealed that, compared with IBD, inflammatory arthritis was not significantly associated with increased in-hospital mortality.^16^

In another US retrospective cohort study, inflammatory arthritis was identified in 20.5% of patients with PG. The prevalence of inflammatory arthritis, along with solid organ malignancies and haematological disorders, was significantly higher among patients with PG aged ≥65 years (*P* < 0.05).^17^

A retrospective cohort study from Argentina also reported arthritis in 16% of patients with PG, including rheumatoid and seronegative arthritis subtypes.^15^

### Vasculitis and Behçets

Two studies evaluated PG in the context of vasculitis, and one study investigated its relationship with Behçets (Table 1; Table S2).

A French retrospective cohort study identified PG in 8 of 743 patients with granulomatosis with polyangiitis (1.1%; *P* = 0.01), indicating a statistically significant association between PG and this form of small-vessel vasculitis.^31^ A large US cross-sectional study of hospitalized patients with PG reported small-vessel vasculitis in 2.5% of cases and found a significantly higher risk of in-hospital mortality compared with patients with PG with IBD (OR 6.0, 95% CI 2.63–13.69).^16^

A retrospective cohort study reported 30% of patients with Behçets (*n* = 6/20), with all cases affecting the lower limbs.^30^

### Organ-specific and metabolic associations

Four studies evaluated the relationship between PG, organ-specific comorbidities and metabolic conditions, with some studies examining both (Table 1; Table S2).

An Israeli retrospective cohort and case–control study reported a bidirectional association between PG and renal disease. Patients with PG had significantly higher risks of chronic renal failure (CRF) (adjusted HR 3.68, 95% CI 2.72–5.97), dialysis (adjusted HR 27.79, 95% CI 3.24–238.14) and other kidney diseases (OKD) (adjusted HR 2.71, 95% CI 1.55–4.74). Similarly, patients with prior CRF (adjusted OR 2.34, 95% CI 1.33–4.11), OKD (adjusted OR 1.69, 95% CI 1.04–2.74) and kidney transplantation (adjusted OR 5.03, 95% CI 1.01–25.12) had increased odds of developing PG.^20^

A Danish nationwide nested case–control study reported higher odds of uncomplicated diabetes (OR 1.92, 95% CI 1.47–2.51), heart failure (OR 2.01, 95% CI 1.50–2.69), moderate-to-severe renal disease (OR 3.24, 95% CI 2.27–4.65) and mild liver disease (OR 2.70, 95% CI 1.69–4.30) in patients with PG compared with control individuals, with all *P*-values <0.001. This subset of patients with PG also demonstrated increased mortality (adjusted HR 2.79, 95% CI 2.57–3.03; *P* < 0.001).^7^ In addition, another Danish retrospective cohort also reported frequent comorbidities among patients with PG, particularly diabetes (25%), and cardiac (20%) and kidney (4.7%) diseases.^14^

In the observational literature, only three studies reported PG subtypes, with the ulcerative form accounting for more than 85% of cases.^14,15,25^

Risk of bias was judged as low to moderate across all included observational studies based on the JBI critical appraisal checklists (Table S2).

## Summary of results (case reports and case series)

A total of 17 case reports and 1 case series (*n* = 8 patients) were identified, describing PG in association with a broad range of systemic diseases not captured by larger observational studies (Table 2; Table S3).

### Systemic disease associations

Vasculitides were the most frequently reported (seven case reports and one case series of eight patients), comprising granulomatosis with polyangiitis, Takayasu arteritis and atypical Cogan syndrome. Most presentations were concurrent with PG diagnosis, although two cases reported PG either years before or after the systemic condition.^34,36^

Autoimmune conditions (six case reports) included systemic lupus erythematosus (SLE), coeliac disease, Evans syndrome and autoimmune hepatitis. In most reports, PG appeared synchronously with the autoimmune disease, although one case described PG presenting 20 years after SLE diagnosis.^40^

Haematological disorders in one case report encompassed haemophagocytic lymphohistiocytosis (HLH), which was identified simultaneously with PG.

Infectious disease associations were rarely described, with one case linking PG with hepatitis C virus infection, diagnosed concurrently with PG.

Other rare associations included Behçets, limited cutaneous systemic sclerosis and a case of microscopic colitis, idiopathic hypereosinophilic syndrome (IHES) and selective IgE deficiency.

### Clinical features

Ulcers most frequently affected the lower limbs, but involvement of the buttocks, trunk, genitalia, periorbital, perioral and upper limbs was also described. Reported subtypes included five ulcerative and one vegetative case, while the remainder were not specified. Pathergy was occasionally documented, and recurrence after remission appeared to be especially common in vasculitis-associated PG.

### Treatment and outcome

Systemic corticosteroids formed the mainstay of therapy, often combined with cyclophosphamide, azathioprine, ciclosporin, methotrexate, intravenous immunoglobulins (IVIg) or hydroxychloroquine. Less conventional approaches were highlighted, including a patient with Takayasu arteritis who responded only to tofacitinib, and an atypical case of Cogan syndrome where hyperbaric oxygen therapy was used adjunctively.^32,36^

Clinical outcomes varied, ranging from complete remission with first-line therapy to multiple relapses necessitating changes in immunosuppressive regimen, and in one case of HLH, a fatal outcome, despite intensive immunosuppression. Recurrence was observed in vasculitis-associated cases, reported in five of seven cases, while two patients achieved remission without relapse.

Most reports were assessed as being at low risk of bias, with a minority judged as being at moderate risk using the JBI checklist (Table S3).

## Discussion

This review synthesizes observational and case-based evidence on systemic disease associations in PG. The results reaffirm established links with IBD, haematological disorders, malignancy and arthritis, and also highlight rarer associations, including vasculitides, autoimmune conditions, connective tissue diseases and infective causes. Importantly, several clinical patterns emerged, including a predominance of the ulcerative PG subtype and relapse-prone disease in vasculitides, which may offer new insights into PG as more than an isolated cutaneous condition.

IBD remains the systemic condition most consistently associated with PG.^1,50^ A large population-based study found that patients with IBD had sixfold increased odds of PG compared with control participants.^24^ In the Danish registry study of over 47 000 patients with IBD and 92 000 control individuals, the odds of PG were markedly elevated (OR 47.5, 95% CI 23.4–96.4).^26^ Although this suggests a robust association, the extreme OR likely reflects the rarity of PG, with wide CIs indicating imprecision. Nevertheless, the finding is directionally consistent with other cohorts and supports the robust link between PG and IBD.^14,16,17,24,28^ Consistently, two large US database studies reported IBD in 26–41% of patients with PG, underscoring its substantial comorbidity burden.^16,17^

The association of PG with CD and UC has long been debated. Earlier case series and observational studies suggested PG was more frequent in UC than in CD.^51,52^ In contrast, more recent registry analyses demonstrate comparable prevalence across both subtypes, indicating that PG is not uniquely associated with either CD or UC.^22,23,28^ This probably reflects shared inflammatory pathways, reinforcing the concept of PG as a cutaneous manifestation of systemic immune dysregulation in IBD rather than being linked to one subtype of IBD.^13,23,53^

Systemic disease activity also appears to play an important role in the development of PG. Historically, PG and IBD activity were thought to follow largely independent courses,^51^ but more recent studies suggest a closer relationship.^54,55^ In our review, several studies reported that PG developed in up to 71% of patients during active IBD compared with those in remission, with resolution of PG following IBD control.^23,25,27^ This dynamic relationship supports that PG occurrence is more tightly linked to IBD activity than previously appreciated.

Haematological disorders represent an important but less common systemic association of PG. Large database studies report prevalence rates of 3–6% in patients with PG, with malignant conditions appearing slightly more frequent than nonmalignant ones.^16,17^ Although less frequent than IBD, their impact may be clinically important. In a US national inpatient study, patients with PG with haematological malignancy had over fourfold increased odds of in-hospital mortality compared with those with IBD (OR 4.31, 95% CI 1.78–10.43).^16^ Although clinically important, wide CIs require cautious interpretation.

In addition to haematological malignancies, MG is emerging as a notable association. A Canadian cohort reported MG in over a quarter of patients with PG, most commonly of the IgA subtype. Importantly, haematological malignancies were substantially more frequent among patients with PG with MG compared with those without (28% vs. 6%; *P* = 0.003), with multiple myeloma accounting for most cases.^18^ This finding suggests that MG may present not only as a concurrent comorbidity but also as a potential marker of increased neoplastic risk in PG populations, a relationship that warrants further study.

The association between PG and SOM remains uncertain. Cohort studies have reported SOM in 6–14% of patients with PG.^14,17^ In contrast, a large Israeli population-based study found no significant bidirectional association between PG and SOM, indicating that their coexistence is more likely coincidental.^19^ This contrasts with earlier literature describing a temporal association and possible paraneoplastic role for SOM in PG.^56,57^

Notably, in the Israeli cohort, patients with PG with concomitant SOM were significantly older at disease onset than those with PG alone: mean (SD) age 71.7 (12.2) years vs. 51.9 (20.7) years (*P* < 0.001). This supports the hypothesis that background, age-­related cancer risk may contribute to the observed coexistence. However, the wide SDs highlight variability and overlap, suggesting that age alone cannot fully explain the association. Overall, the current evidence does not support a direct causal link.^19^

The association between PG and inflammatory arthritis appears to be relatively frequent, with prevalence estimates ranging from 7% to 21% across observational studies.^15–17^ Rheumatoid arthritis and seronegative arthritides have been reported among patients with PG, indicating that PG may accompany a broader spectrum of immune-mediated joint disease rather than a single form of arthropathy. Notably, this broader association was already recognized in earlier literature.^58,59^

Mortality outcomes in PG and arthritis have been inconsistently reported. In a large US inpatient cohort, an in-hospital mortality risk of 2% over 8 years was observed, and inflammatory arthritis was not significantly associated with increased mortality compared with IBD.^16^ In contrast, a retrospective review of 103 patients with PG reported a substantially higher overall mortality rate of 16%, in which rheumatological comorbidities were common.^53^ These discrepancies probably reflect differences in the study design, population and follow-up. Further research is needed to clarify the prognostic impact of inflammatory arthritis in PG.

The relationship between PG and vasculitic disorders appears uncommon but clinically relevant. In a French retrospective cohort, PG was identified in 1.1% of patients with granulomatosis with polyangiitis, representing a rare but statistically significant association (*P* = 0.01).^31^ In addition, a large US cross-sectional study reported vasculitis in 2.5% of hospitalized patients with PG and demonstrated a sixfold higher risk of in-hospital mortality compared with patients with PG with IBD (OR 6.0, 95% CI 2.63–13.69).^16^ While this suggests a trend towards increased mortality, the wide CIs indicate unstable estimates, probably due to small sample size. Larger studies are needed to confirm the risk.

Behçets has also been reported in association with PG. In one cohort, PG was observed in 30% of patients with Behçets, predominantly affecting the lower limbs.^30^ The predilection for the lower limbs may reflect pathergy phenomena, as PG and Behçets ulcers are often precipitated by trauma to dependent sites.^60–63^ The coexistence of PG and Behçets poses diagnostic challenges due to overlapping ulcerative features and may reflect shared neutrophilic and pathergy-driven mechanisms.^63–65^ Further studies are required to assess whether Behçets should be regarded as a systemic association of PG or a coincidental overlap.

Renal disease has emerged as the most consistently reported organ-specific association of PG. An Israeli bidirectional cohort and case–control study demonstrated that patients with PG had markedly increased risks of CRF, dialysis and OKD, while prior CRF and kidney transplantation were also associated with subsequent PG.^20^ The consistency of findings strengthens the evidence for an accurate association despite some limitations in data quality. Supporting this, a Danish nationwide study similarly demonstrated renal involvement, and also identified increased risks of heart failure and mild liver disease, suggesting that PG may be associated with a broader systemic pattern.^7^

Diabetes appears to be the most frequent metabolic comorbidity associated with PG.^14,15^ In a Danish nationwide study, patients with PG had almost double the odds of uncomplicated diabetes compared with control individuals (OR 1.92, 95% CI 1.47–2.51; *P* < 0.001).^7^ Another retrospective Danish cohort also demonstrated diabetes as highly prevalent, affecting 25% of patients with PG.^14^ Importantly, patients with PG with metabolic and organ-related comorbidities also had substantially higher mortality, with almost a threefold increased risk of death compared with control individuals.^7^ Although these associations appear to be clinically relevant, it remains uncertain whether diabetes represents a true pathogenic link with PG or reflects coincidental co-­occurrence due to background multi­morbidity in older patient populations.

Most observational studies were judged to be at low-to-­moderate risk of bias. This supports the robustness of the identified associations, although heterogeneity in study design, diagnostic criteria and adjustment for confounders may have influenced effect estimates.

Evidence from case reports and series expands the spectrum of systemic conditions associated with PG beyond those captured in larger observational cohorts. Vasculitides were the most frequently described, including granulomatosis with polyangiitis, Takayasu arteritis and atypical Cogan syndrome. Vasculitis-associated PG also often demonstrated a relapse-prone course, suggesting the need for long-term monitoring and sustained immunosuppression. PG usually appeared concurrently with vasculitis, although in some cases the onset was separated by several years, reflecting temporal variability.

Autoimmune diseases were represented, including Behçets, SLE, coeliac disease, Evans syndrome and autoimmune hepatitis. Most appeared concurrently with PG, although a delayed onset was occasionally reported, such as a case of PG occurring decades after SLE.^40^ Ulcerative PG was the predominant subtype within these autoimmune associations. Haematological, connective ­tissue disease, infectious and other rare associations were infrequently described. These include HLH (one case with a fatal ­outcome), limited cutaneous systemic sclerosis, hepatitis C virus infection and a case of microscopic colitis with IHES and selective IgE deficiency, highlighting the heterogeneity of the systemic contexts in which PG can occur.^46–49^

Clinical features were consistent with observational data, with lower limb involvement being the most frequent. However, ­atypical sites such as buttocks, trunk, genitalia, upper limbs, periorbital and perioral areas were also described. Pathergy was occasionally noted, and recurrence following remission was common in vasculitis-associated PG. Treatments were heterogeneous, with systemic corticosteroids as the mainstay, often combined with immunosuppressants such as ciclosporin, cyclophosphamide, azathioprine, methotrexate and hydroxychloroquine. Immuno­modulatory approaches included IVIg. Less conventional ­interventions were outlined in one case of refractory Takayasu arteritis–associated PG that achieved remission with tofacitinib, suggesting a potential role for Janus kinase inhibition in neutrophilic dermatoses, and adjunctive hyperbaric oxygen therapy was used successfully with cyclophosphamide in a case of atypical Cogan syndrome.^33,37^ Outcomes ranged from complete remission after first-line therapy to multiple relapses and, in rare cases, fatality despite intensive treatment.

Collectively, these cases illustrate the remarkable heterogeneity of PG’s systemic associations. While these observations broaden the clinical spectrum and generate hypotheses for shared autoinflammatory and immunological mechanisms, their interpretation is constrained by small numbers, variable reporting quality and publication bias. Although most reports were judged to be at low risk of bias using the JBI checklist, case reports and series remain inherently biased and should be considered as ­hypothesis-generating rather than confirmatory.

This review was restricted to publications written in English, limiting international data collection. The included ­studies were heterogenous in design, diagnostic criteria and reporting, which may reduce comparability and precluded meta-analysis. To address this, each study underwent independent risk-of-bias assessment by two reviewers. In addition, the case-based literature carries a risk of publication bias, as atypical or more severe presentations are more likely to be reported. However, it also provides valuable clinical insights where observational evidence is lacking. Finally, restricting inclusion to studies from 2015 onwards may have omitted older associations but allowed us to focus on the most recent evidence and PG links.

In summary, PG is rarely an isolated skin disorder and is ­frequently linked with systemic diseases. Well-established associations with IBD, haematological disorders and arthritis are re­­affirmed, while additional links with vasculitis, organ-specific and metabolic disorders broaden its spectrum. The predominance of the ulcerative subtype and relapse-prone vasculitis-associated PG provide new insights. Larger prospective studies are needed to clarify causality and guide management.

## Supplementary Material

vzag037_Supplementary_Data

## Data Availability

The dataset presented in this study is available on request from the corresponding author.
