# Supplementary material for: Systemic associations of pyoderma gangrenosum: a systematic review
Source: Skin Health Dis. 2026 May 26;6(4):393–405. doi: 10.1093/skinhd/vzag037 (PMC13425086; doi:10.1093/skinhd/vzag037)
Supplement: vzag037_Supplementary_Data [file vzag037_supplementary_data.zip › SHD-2025-0388.r2 Supplementary Table 2.docx]

| **Supplementary Table 2. Full** **Summary of Observational Studies Assessing Systemic Associations of Pyoderma Gangrenosum** | | | | | | | | | |
| --- | --- | --- | --- | --- | --- | --- | --- | --- | --- |
| **Author** | **Study Design** | **Population**  **Studied** | **Control** | **Disease**  **Association** | **Key findings** | **Timing of systemic disease diagnosis relative to PG** | **Systemic Disease Activity at PG Presentation** | **Novelty** | **Risk of Bias**  **(JBI)** |
| Schosler, 2021 [Denmark]^25^ | Retrospective cohort study | PG (N=64) | No | Multiple | IBD (28.1%), diabetes type II (21.9%), cardiac (20.3%), malignancy after PG (14.1%), RA (12.5%), thyroid disease (12.5%), other autoimmune diseases (12.5%), gout (7.8%), haematological (7.8%), malignancy before PG (6.3%), kidney disease (4.7%), HS (4.7%), diabetes type I (3.1%), parkinsonism (3.1%), haemophagocytosis (1.6%).  Types of PG: ulcerative (85.9%), peristomal (6.3%), bullous (3.1%), vegetative (3.1%), pustular (1.6%) | Before and after PG diagnosis. | Active | Mixed | Moderate |
| Abdallah, 2021 [Denmark]^7^ | Nationwide registry nested case–control study | PG (N=1604) | Yes, 16039 | Multiple | 1023 of 1604 had a co-morbidity (64%) at time of control vs 33% in the control group.  Adjusted OR for co-morbidity comparing patients with PG to controls, P-value:   - Diabetes without complication 1.92 (1.47-2.51), <0.001 - Heart failure or cardiomyopathy 2.01 (1.50-2.69), <0.001 - Hemiplegia or paraplegia 6.48 (3.78-1.10), <0.001 - Hidradenitis or acne 4.15 (1.77-9.72), 0.001 - Lymphoma 2.92 (1.51-5.65), 0.001 - IBD 6.51 (4.24-10.01), <0.001 - Leukaemia 7.50 (4.41-12.75), <0.001 - Mild liver disease 2.70 (1.69-4.30), <0.001 - Moderate-to-severe renal disease 3.24 (2.27-4.65), <0.001 - Osteoporosis 1.57 (1.22-2.02), <0.001 - Peripheral vascular disease 3.33 (2.71-4.08), <0.001 - Rheumatic disease 2.56 (2.17-3.03), <0.001   Adjusted HR for mortality with 95% CI, P-value:  2.79 (2.57-3.03), <0.001. | At the time of PG diagnosis. | Not reported | Mixed | Low |
| Vacas, 2017 [Argentina]^22^ | Retrospective cohort study | PG (N=31) | No | Multiple | 23 of 31 with PG (74%) had associated systemic disease: IBD 10 (32%), haematological malignancies 7 (22%), rheumatoid or seronegative arthritis 5 (16%), diabetes 5 (16%), cocaine consumption 1 (3%). | 4 of 7 diagnosed IBD before PG; 3 diagnosed after PG. | Not reported | Well known | Moderate |
| Kaffenberger, 2018 [USA]^20^ | Retrospective cross-sectional study | PG (N=31885) | No | Multiple | Co-morbidities: IBD 8453 (26.5%), inflammatory arthritis 2345 (7.35%), vasculitis and HSP 795 (2.49%), haematological malignancy and dyscrasia 886 (2.78%); others 19405 (60.86%).  From multivariable logistic regression modelling, OR for mortality vs. IBD with 95% CI   - Small vessel vasculitis 6.0, (2.63–13.69), - Haematological dyscrasias 4.31, (1.78–10.43), - Others 2.23, (1.21–4.11), - Sepsis 12.43, (8.65–17.86), - Increasing age 1.04, (1.03–1.05). | Not reported | Not reported | Well known | Low |
| Ashchyan, 2018 [USA]^19^ | Retrospective cohort | PG (N=356) | No | Multiple | 238 of 356 with PG (66.9%) had co-morbidities:   - IBD: 146 patients (41%): CD 92 (25.8%), UC: 55 (15.4%). - Arthritis: 73 (20.5%). - Malignant neoplasms: 44 (12.4%): solid organ malignancies 23 (6.5%) and haematological malignancies: 21 (5.9%). - Haematological disorders (nonmalignant): 17 (4.8%).   Age associations: IBD was significantly more common in patients aged <65 years (p<0.001); and arthritis, solid organ malignancies, and haematological disorders (including malignancies) were more common in patients aged ≥65 (p < 0.05**)** | Not reported | Not reported | Well-known | Moderate |
| Saeidi, 2024 [Canada]^24^ | Retrospective cohort study | PG (N=106) | No | Monoclonal gammopathy [Haematological] | 29 of 106 with PG (27%) had monoclonal gammopathy (MG).  Commonest subtype: IgA (41%), IgG (28%) and biclonal IgA/IgG (14%).  Cancer developed in significantly more patients with PG with gammopathy than without (28% vs. 6%, *p* = 0.003), predominantly hematologic malignancies (especially multiple myeloma). Among subtypes, IgG MG had the highest proportion of cancer (50%). | Not reported | Not reported | Well-known | Moderate |
| Kridin, 2021 [Israel]^26^ | Retrospective cohort + case-control | PG (N=302) | Yes, 1497 | Solid organ malignancy | No significant bidirectional association between PG and SOM was found.   - Risk of SOM after PG: (adj HR 1.02; 95% CI 0.52–2.02) - Risk of PG after SOM: adj OR 0.85 (95% CI 0.51–1.41; p = 0.522)   Patients with coexistent PG and SOM were significantly older at PG onset compared to those with PG alone (mean (SD) age 71.7 (12.2) years vs. 51.9 (20.7) years ; *P* < 0.001) | Not reported | Not reported | Well-known | Cohort - Low Case-control - Low |
| Kridin, 2021 [Israel]^29^ | Retrospective cohort + case-control | PG (N=302) | Yes, 1497 | Chronic renal comorbidities (CRC) | A bidirectional association was observed between PG and several renal comorbidities. In the retrospective cohort analysis, patients with PG had an increased risk of:   - Chronic renal failure (CRF) (adj HR 3.68; 95% CI 2.72–5.97) - Dialysis (adj HR 27.79; 95% CI 3.24–238.14) - Other kidney diseases (OKD) (adj HR 2.71; 95% CI 1.55–4.74); No significant association with kidney transplantation (KT).   In the case–control analysis**,** patients with prior renal comorbidities had higher odds of PG:   - CRF (adj OR 2.34; 95% CI 1.33–4.11) - KT (adj OR 5.03; 95% CI 1.01–25.12) - OKD (adj OR 1.69; 95% CI 1.04–2.74); No significant association for prior dialysis.   Patients with PG and CRC were older than those without CRC (mean age >60 years, p < 0.05). | Not reported | Not reported | Well-known | Cohort - Low Case-control - Low |
| Juliao-Baños, 2020 [Colombia]^35^ | Retrospective cohort | IBD (N=744) | No | PG | 8 of 744 with IBD (1.1%) had PG: 7 of 544 with UC (1.2%) and 1 of 200 with CD (0.50%) No effect estimate provided; descriptive prevalence only | Not reported | Not reported | Well-known | Moderate |
| Jonaityte, 2024 [Lithuania]^18^ | Prospective cross-sectional | IBD (N=162) | No | PG | 6 of 162 with IBD (3.7%) had PG: with 5 of 117 UC (4.3%) and 1 of 45 CD (2.2%).  No statistically significant difference between UC and CD (p = 0.877). No association with IBD duration (p > 0.05). | All PG cases developed at or after IBD diagnosis | Not reported | Well-known | Moderate |
| Ghani, 2024 [Pakistan]^17^ | Retrospective Cross-sectional study | IBD (N=250) | No | PG | 14 of 250 with IBD (5.6%) had PG; no significant difference between CD (6.4%) and UC (4.5%) (p = 0.45). PG significantly more common in active disease (45%) than remission (18%) (p < 0.001). | Not reported | Active disease (flare) | Well-known | Moderate |
| Waljee, 2020 [USA]^14^ | Retrospective cross-sectional study | Single centre: IBD (N=6225).  National:  IBD (N=80907) | Yes. Single centre: 31125.  National: 404535 | PG | Single centre: 44 of 6225 IBD (0.71%) had PG, 10 of 31125 controls (0.03%) had PG; OR 22.15, p-value 0.0001.  National: 607 of 80907 IBD (0.8%) had PG, 492 of 404535 controls (0.1%) had PG; OR 6.2, p-value 0.0001 | Not reported | Not reported | Well-known | Moderate |
| Giraudo, 2020 [Argentina]^21^ | Retrospective observational study | IBD (N=444) | No | PG | 7 of 444 had PG (1.6%): 5 had established IBD, 2 were concomitantly diagnosed with both IBD and PG.  One patient developed PG one year after colectomy. | Before and concurrent with PG diagnosis | 1 mild, 4 moderate, 1 severe, 1 remission. | Well-known | Moderate |
| Halling, 2017 [Denmark]^15^ | Nationwide retrospective cross-sectional study. | IBD (N=47325) | Yes, 92839 | PG | 193 of 47325 with IBD had PG, with OR 47.5, 95% CI (23.4-96.4).  8 of the 92839 controls had PG. | Not reported | Not reported | Well known | Moderate |
| Padhi, 2023 [India]^22^ | Prospective cohort study | UC (N=112) | Yes, 100 | PG | 1 of 112 with UC (0.9%) had PG, which disappeared within a month of treatment of the UC. | Not reported | Not reported | Well-known | Moderate |
| Yang, 2018 [Korea]^16^ | Nationwide retrospective cross-sectional study | CD (N=13925)  UC (N= 29356) | Yes, 1127261 | PG | 32 of 13925 with CD (0.2%) had PG: 10M and 22F.  34 of 29356 with UC (0.1%) had PG: 13M and 21F.  341 of 1127261 controls (0.0%) had PG: 95M and 246F.  Standardised prevalence ratio (95% CI) for the CD group was 4.43 (1.36-7.50) and the UC group was 4.36 (2.50-6.23). | Not reported | Not reported | Well known | Moderate |
| Lee, 2016 [Korea]^36^ | Retrospective cohort study | MDS with AIMs(N=67) | Yes, 134 MDS without AIMs | PG | 2 of 67 with MDS had PG. | Not reported | Not reported | Rare | Moderate |
| Bugaut, 2023 [France]^28^ | Retrospective cohort study | Behçet’s disease (N=20) | No | PG | 6 of 20 with BD (30%) had PG and all involved the lower limbs. | Not reported | Not reported | Rare | Moderate |
| Frumholtz, 2017 [France]^27^ | Retrospective cohort | AAV (N=1553)  [Vasculitis] | No | PG | 8 of 1553 with AAV had PG and they were all GPA; nil in EGPA and MPA, P-value=0.01. | Not reported | Not reported | Rare | Moderate |
| *Legend for Supplementary Table 2: full extracted data from observational studies describing systemic associations of pyoderma gangrenosum (PG). Timing of PG relative to systemic disease refers to whether PG developed before, concurrently with, or after diagnosis of the systemic condition. Abbreviations: PG, pyoderma gangrenosum; IBD, inflammatory bowel disease; RA, rheumatoid arthritis; JBI, Joanna Briggs institute; HR, hazard ratio; HSP, Henoch–Schönlein purpura; OR, odds ratio; CI, confidence interval; CD, Crohn’s disease; UC, ulcerative colitis; SOM, solid organ malignancy; adj, adjusted; AIMs, auto-immune manifestations; MDS, myelodysplastic syndrome; BD, Behçet’s disease; AAV, Anti-Neutrophil Cytoplasmic Antibodies-associated vasculitides; GPA, granulomatosis with polyangiitis; EGPA, eosinophilic granulomatosis with polyangiitis; MPA, microscopic polyangiitis.* | | | | | | | | | |
