# Supplementary material for: Systemic associations of pyoderma gangrenosum: a systematic review
Source: Skin Health Dis. 2026 May 26;6(4):393–405. doi: 10.1093/skinhd/vzag037 (PMC13425086; doi:10.1093/skinhd/vzag037)
Supplement: vzag037_Supplementary_Data [file vzag037_supplementary_data.zip › SHD-2025-0388.R2 Supplementary Table 3.docx]

| **Supplementary Table 3. Full Summary of case reports and case series on pyoderma gangrenosum with systemic disease associations.** | | | | | | | | | | | | | |
| --- | --- | --- | --- | --- | --- | --- | --- | --- | --- | --- | --- | --- | --- |
| **Author** | **Population size** | **Age** | **Sex** | **Systemic Disease** | **Systemic Disease Category** | **Timing of Systemic Disease Diagnosis Relative to PG** | **Site(s)**  **of Involvement** | **PG Subtypes** | **Pathergy Present? (Yes/No)** | **Recurrence (Yes/No)** | **Treatment + Outcome** | **Novelty** | **Risk of Bias (JBI)** |
| Chua, 2018 [UK]  (Case report)^37^ | 1 | 49 | F | Atypical Cogan's syndrome | Vasculitis | Concurrent | Both shins | Not reported | Not reported | Yes | Multiple therapies failed; good response to CYC | Rare | Low |
| Nadarajah, 2025 [UK]  (Case report)^34^ | 1 | 75 | F | Atypical Cogan's syndrome | Vasculitis | Before | R Buttock, L hip and buttock | Not reported | Yes | No | Good response to corticosteroids + CYC + hyperbaric oxygen therapy | Rare | Low |
| de Boysson, 2016 [France]  (Case series)^31^ | 8 | Range  30-62 | 5 M, 3 F | GPA | Vasculitis | 4 concurrent, 2 before PG, 2 after PG. | Abdo, lower limbs, trunk, back | Not reported | Not reported | Yes | All responded to corticosteroids ± immunosuppressants (CYC, AZA, IVIG, CsA) | Rare | Low |
| Malinowska, 2020 [Poland]  (Case report)^38^ | 1 | 41 | M | GPA | Vasculitis | Concurrent | L shin | Not reported | Yes | No | Partial response to corticosteroids + CsA + pentoxifylline; good response to corticosteroids + CYC, with AZA for maintenance | Rare | Moderate |
| Barrera-Vargas 2015 [Mexico]  (case report)^30^ | 2 | 21 & 26 | 1F  1M | Takayasu’ s arteritis | Vasculitis | 1.Five years after PG.  2.Eight years after PG. | 1. Head, neck, arms, lower limbs. | Not reported | No | Yes | 1.Partial response to prednisone, thalidomide.  2. Good response to prednisone, CsA, thalidomide, dapsone, AZA. | Rare | Moderate |
| Bhowmick, 2023 [India]  (Case report)^33^ | 1 | 22 | F | Takayasu arteritis | Vasculitis | Concurrent | Bilateral upper limbs and lower limbs. | Not reported | Yes | Yes | Trialled corticosteroids, MTX, colchicine, tocilizumab, antibiotics; responded only to tofacitinib | Rare | Moderate |
| Loetscher, 2016 [Switzerland]  (Case report)^39^ | 1 | 43 | F | Takayasu arteritis | Vasculitis | Concurrent | Bilateral lower limbs. | Not reported | Not reported | Yes | Good response to corticosteroids + CsA + MTX | Rare | Moderate |
| Ozuguz, 2015 [Turkey]  (Case report)^40^ | 1 | 33 | F | BD | Autoinflammatory/Autoimmune | Concurrent | R labium minor | Vegetative | Not reported | No | No response to colchicine, good response to corticosteroids | Rare | Low |
| Pourbagherian, 2023 [Iran]  (Case report)^32^ | 1 | 55 | F | SLE | Autoimmune | Before PG by 20 years | Anterior and lateral R leg | Not reported | Not reported | No | Good response to corticosteroids + CYC + Hydroxychloroquine + mycophenolate mofetil. | Rare | Low |
| Lebrun 2018 [France]  (Case report)^41^ | 2 | 32 & 37 | 2F | SLE | Autoimmune | 1. One year before PG.  2. Ten years before PG. | 1: Inner canthus L eye, L corner of lips, cervical.  2. Posterior legs | Ulcerative for both | Not reported | No | 1.Complete response to corticosteroids.  2. Failure (side effects) with dapsone, colchicine, low dose corticosteroid. Complete response with corticosteroids and MTX. | Rare | Low |
| Frioui, 2022 [Tunisia]  (Case report)^42^ | 1 | 52 | F | Coeliac disease | Autoimmune | concurrent | Anterior L shin | Ulcerative | Not reported | No | Good response to corticosteroids | Rare | Low |
| Yang 2018 [Taiwan]  (Case report)^43^ | 1 | 33 | M | Evans syndrome | Autoimmune | Seven years before PG | Scrotum | Ulcerative | No | No | Complete response to IV methylprednisolone, then prednisolone and AZA. | Rare | Low |
| Dantas 2017 [Brazil]  (Case report)^44^ | 1 | 25 | F | Autoimmune hepatitis | Autoimmune | Concurrent | Bilateral lower limbs | Ulcerative | Not reported | No | Complete response with prednisolone; topical non-corticosteroid treatment and dressings. | Rare | Low |
| Androutsakos 2015 [Greece]  (Case report)^45^ | 1 | 19 | F | Autoimmune hepatitis | Autoimmune | Four years before PG | Bilateral legs | Not reported | Possible | No | Complete response with prednisolone, CsA, MTX | Rare | Low |
| Kaur, 2022 [India]  (Case report)^46^ | 1 | 43 | F | Hepatitis C Infection | Infectious | Concurrent | Right lower limbs and L buttock | Not reported | No | No | Good response to corticosteroids + antibiotics. | Rare | Moderate |
| Skopsis 2021 [USA]  (Case report)^47^ | 1 | 65 | F | Limited cutaneous systemic sclerosis | Connective tissue disease | Concurrent | L index finger | Not reported | Not reported | No | Complete response to IV methylprednisolone, then prednisolone and topical clobetasol 0.05%. | Rare | Low |
| Riyaz, 2015 [India]  (Case report)^48^ | 1 | 27 | M | Microscopic colitis, IHES, Selective IgE deficiency | Gastrointestinal (non-IBD), haematological, primary immunodeficiency | concurrent | L lower leg | Not reported | Yes | No | Good response to corticosteroids. | Rare | Low |
| Opalińska, 2021 [Poland]  (Case report)^49^ | 1 | 35 | M | HLH | Haematological (Nonmalignant) | Concurrent | Bilateral upper limbs and lower limbs. | Not reported | Not reported | Yes | Multiple systemic therapies trailed (corticosteroids, CsA, IVIG, anakinra); fatal outcome due to sepsis and multiorgan failure. | Rare | Low |
| *Legend for Supplementary Table 3: full extracted data from case reports and case series describing rare or under-recognised systemic associations of pyoderma gangrenosum (PG). Treatments listed refer specifically to those administered for PG, and treatment outcomes reflect the clinical response as described in the source reports. Timing of PG relative to systemic disease refers to whether PG developed before, concurrently with, or after diagnosis of the systemic condition. Abbreviations: PG, pyoderma gangrenosum; JBI, Joanna Briggs institute; F, female; M, male; CYC, cyclophosphamide; R, right; L, left; GPA, granulomatosis with polyangiitis; AZA, azathioprine; IVIG, intravenous immunoglobulins; CsA, ciclosporin; MTX, methotrexate; BD, Behçet’s disease; SLE, systemic lupus erythematosus; IHES, idiopathic hypereosinophilic syndrome; IBD, inflammatory bowel disease.* | | | | | | | | | | | | | |
