# Supplementary material for: Systemic associations of pyoderma gangrenosum: a systematic review
Source: Skin Health Dis. 2026 May 26;6(4):393–405. doi: 10.1093/skinhd/vzag037 (PMC13425086; doi:10.1093/skinhd/vzag037)
Supplement: vzag037_Supplementary_Data [file vzag037_supplementary_data.zip › SHD-2025-0388.R2 Supplimentary Table 1.docx]

| **Supplementary Table 1: Search Strategy for Systematic Review** | | | | |
| --- | --- | --- | --- | --- |
| **Database** | **Platform/Interface** | **Search Terms** | **Limits/Filters Applied** | **Date of Search** |
| PubMed | NCBI | (Pyoderma Gangrenosum[MeSH Terms] OR "pyoderma gangrenosum"[Title/Abstract]) AND  (  Lung[MeSH Terms] OR bronchial OR alveolar OR thoracic OR pulmonary OR respiratory OR "interstitial lung disease" OR pleuritis OR bronchiectasis OR  Vascular Diseases[MeSH Terms] OR heart OR myocardium OR endocarditis OR vasculitis OR arrhythmia OR ischemia OR cardiovascular OR cardiac OR  musculoskeletal OR arthritis OR myalgia OR tenosynovitis OR joint OR rheumatic OR arthralgia OR spondyloarthritis OR enthesitis OR "rheumatoid arthritis" OR  Hematologic Diseases[MeSH Terms] OR leukemia OR "myelodysplastic syndrome" OR lymphoma OR "multiple myeloma" OR "hematologic malignancy" OR "bone marrow" OR anemia OR "immune dysregulation" OR  Nervous System[MeSH Terms] OR neuropathy OR encephalopathy OR seizures OR brain OR paresthesia OR  oral cavity OR gingival OR pharynx OR nasal OR sinusitis OR rhinosinusitis OR buccal OR laryngeal OR  Eye Diseases[MeSH Terms] OR conjunctivitis OR keratitis OR scleritis OR ophthalmic OR ocular OR  lymph OR lymphatic OR lymphadenopathy OR "immune-mediated" OR immune OR  Reproductive System[MeSH Terms] OR genital OR cervix OR uterus OR urethra OR vagina OR penis OR  Gastrointestinal Diseases[MeSH Terms] OR "Inflammatory Bowel Diseases" OR Crohn’s OR colitis OR "liver disease" OR cholangitis OR hepatitis OR pancreatitis OR biliary OR hepatomegaly OR spleen OR splenic OR splenomegaly OR  Kidney[MeSH Terms] OR nephropathy OR "renal failure" OR proteinuria OR "urinary tract" OR urological OR urogenital OR  Fatigue[MeSH Terms] OR Fever[MeSH Terms] OR Weight Loss[MeSH Terms] OR "night sweats" OR "constitutional symptoms" OR  systemic OR extracutaneous OR comorbidities OR multisystem OR visceral  )  AND ("2015/01/01"[Date - Publication] : "3000"[Date - Publication]) | English language; Humans; Last 10 years | 24/05/2025 |
| Scopus | Elsevier | TITLE-ABS("pyoderma gangrenosum") AND  (  TITLE-ABS(lung OR bronchial OR alveolar OR thoracic OR pulmonary OR respiratory OR "interstitial lung disease" OR pleuritis OR bronchiectasis) OR  TITLE-ABS("vascular disease*" OR heart OR myocardium OR endocarditis OR vasculitis OR arrhythmia OR ischemia OR cardiovascular OR cardiac) OR  TITLE-ABS(musculoskeletal OR arthritis OR myalgia OR tenosynovitis OR joint OR rheumatic OR "joint pain" OR "rheumatoid arthritis" OR spondyloarthritis OR enthesitis) OR  TITLE-ABS("hematologic disease*" OR leukemia OR "myelodysplastic syndrome*" OR lymphoma OR "multiple myeloma" OR "hematologic malignancy" OR myelodysplasia OR anemia OR "bone marrow" OR "immune dysregulation") OR  TITLE-ABS("nervous system" OR neuropathy OR encephalopathy OR seizures OR brain OR paresthesia) OR  TITLE-ABS("oral cavity" OR gingival OR pharynx OR nasal OR sinusitis OR rhinosinusitis OR buccal OR laryngeal) OR  TITLE-ABS("eye disease*" OR conjunctivitis OR keratitis OR scleritis OR ophthalmic OR ocular) OR  TITLE-ABS(lymph OR lymphatic OR lymphadenopathy OR "immune-mediated" OR immune) OR  TITLE-ABS("reproductive system" OR genital OR cervix OR uterus OR urethra OR vagina OR penis) OR  TITLE-ABS("gastrointestinal disease*" OR "inflammatory bowel disease*" OR Crohn's OR colitis OR "liver disease*" OR cholangitis OR hepatitis OR pancreatitis OR biliary OR hepatomegaly OR spleen OR splenic OR splenomegaly) OR  TITLE-ABS(kidney OR nephropathy OR "renal failure" OR proteinuria OR "urinary tract" OR urological OR urogenital) OR  TITLE-ABS(fatigue OR fever OR "weight loss" OR "night sweats" OR "constitutional symptoms") OR  TITLE-ABS(systemic OR extracutaneous OR comorbidities OR multisystem OR visceral)  )  AND LANGUAGE(english)  AND PUBYEAR > 2014 | English language; Humans; Last 10 years | 24/05/2025 |
| EMBASE | Ovid | 1. pyoderma gangrenosum.ti,ab.  2. lung/ OR bronchial.ti,ab. OR alveolar.ti,ab. OR thoracic.ti,ab. OR pulmonary.ti,ab. OR respiratory.ti,ab. OR interstitial lung disease.ti,ab. OR pleuritis.ti,ab. OR bronchiectasis.ti,ab.  3. vascular disease/ OR heart/ OR myocardium/ OR endocarditis/ OR vasculitis/ OR arrhythmia.ti,ab. OR ischemia.ti,ab. OR cardiovascular.ti,ab. OR cardiac.ti,ab.  4. musculoskeletal system/ OR arthritis.ti,ab. OR myalgia.ti,ab. OR tenosynovitis.ti,ab. OR joint.ti,ab. OR rheumatic.ti,ab. OR arthralgia.ti,ab. OR rheumatoid arthritis.ti,ab. OR spondyloarthritis.ti,ab. OR enthesitis.ti,ab.  5.hematologic disease/ OR leukemia/ OR myelodysplastic syndrome/ OR lymphoma/ OR multiple myeloma/ OR hematologic malignancy.ti,ab. OR myelodysplasia.ti,ab. OR bone marrow.ti,ab. OR anemia.ti,ab.  6. nervous system/ OR neuropathy.ti,ab. OR encephalopathy.ti,ab. OR seizures.ti,ab. OR brain.ti,ab. OR paresthesia.ti,ab.  7. oral cavity/ OR gingiva/ OR pharynx/ OR nasal cavity/ OR sinusitis.ti,ab. OR rhinosinusitis.ti,ab. OR buccal.ti,ab. OR laryngeal.ti,ab.  8. eye disease/ OR conjunctivitis.ti,ab. OR keratitis.ti,ab. OR scleritis.ti,ab. OR ophthalmic.ti,ab. OR ocular.ti,ab.  9. reproductive system/ OR genital.ti,ab. OR cervix.ti,ab. OR uterus.ti,ab. OR urethra.ti,ab. OR vagina.ti,ab. OR penis.ti,ab.  10. gastrointestinal disease/ OR inflammatory bowel disease/ OR Crohn disease/ OR colitis/ OR liver disease/ OR cholangitis/ OR hepatitis/ OR pancreatitis/ OR biliary.ti,ab. OR hepatomegaly.ti,ab. OR spleen/ OR splenic.ti,ab. OR splenomegaly.ti,ab.  11. kidney/ OR nephropathy.ti,ab. OR renal failure/ OR proteinuria.ti,ab. OR urinary tract/ OR urological.ti,ab. OR urogenital.ti,ab.  12. fatigue.ti,ab. OR fever.ti,ab. OR weight loss.ti,ab. OR night sweats.ti,ab. OR constitutional symptoms.ti,ab.  13. or/2-12  14. 1 and 13  15. limit 14 to (english language and humans and yr="2015 -Current") | English language; Humans; Last 10 years | 24/05/2025 |
