# Supplementary material for: Systemic associations of pyoderma gangrenosum: a systematic review
Source: Skin Health Dis. 2026 May 26;6(4):393–405. doi: 10.1093/skinhd/vzag037 (PMC13425086; doi:10.1093/skinhd/vzag037)
Supplement: vzag037_Supplementary_Data [file vzag037_supplementary_data.zip › Supporting Information SHD-2025-0388.R2 PRISMA Flow.docx]

**Supporting Information**

**Supplementary Figure 1**

**PRISMA 2020 flow diagram for new systematic reviews which included searches of databases and registers only**

**Identification of studies via databases**

Records removed *before screening*:

Duplicate records removed

(n = 1418)

Records identified from PubMed, Embase and Scopus:

Total (n = 3629)

**Identification**

Records screened (titles and abstracts)

(n = 2211)

Records excluded

(n = 2105)

Reports sought for retrieval

(n = 106)

Reports not retrieved

(n = 8) as no full-text PDF available

**Screening**

Reports excluded after full-text review (n = 61)

Lack of clinical details (n = 16)

Systemic disease occurred years after PG (n = 8)

Includes paediatric population (n = 27)

Includes trauma-induced PG (n = 5)

Includes drug-induced PG

(n = 1)

PG mimicker (n = 1)

Wrong study design (n = 1)

Duplication (n = 2)

etc.

Reports assessed for eligibility

(n = 98)

Studies included in review

(n = 37)

**Included**

Source: Page MJ, et al. BMJ 2021;372:n71. doi: 10.1136/bmj.n71.

This work is licensed under CC BY 4.0. To view a copy of this license, visit <https://creativecommons.org/licenses/by/4.0/>
